# Molecular Modeling and Design Studies of Purine Derivatives as Novel CDK2 Inhibitors

**DOI:** 10.3390/molecules23112924

**Published:** 2018-11-09

**Authors:** Gaomin Zhang, Yujie Ren

**Affiliations:** School of Chemical and Environmental Engineering, Shanghai Institute of Technology, Shanghai 201418, China; gaomin.zhang@hotmail.com

**Keywords:** CDK2, 3D-QSAR, virtual screening, molecular docking, lead compound optimization, molecular dynamics

## Abstract

Cyclin-dependent kinase 2 (CDK2) is a potential target for treating cancer. Purine heterocycles have attracted particular attention as the scaffolds for the development of CDK2 inhibitors. To explore the interaction mechanism and the structure–activity relationship (SAR) and to design novel candidate compounds as potential CDK2 inhibitors, a systematic molecular modeling study was conducted on 35 purine derivatives as CDK2 inhibitors by combining three-dimensional quantitative SAR (3D-QSAR), virtual screening, molecular docking, and molecular dynamics (MD) simulations. The predictive CoMFA model (q^2^ = 0.743, $r_{\mathrm{pred}}^{2}$ = 0.991), the CoMSIA model (q^2^ = 0.808, $r_{\mathrm{pred}}^{2}$ = 0.990), and the Topomer CoMFA model (q^2^ = 0.779, $r_{\mathrm{pred}}^{2}$ = 0.962) were obtained. Contour maps revealed that the electrostatic, hydrophobic, hydrogen bond donor and steric fields played key roles in the QSAR models. Thirty-one novel candidate compounds with suitable predicted activity (predicted pIC_50_ > 8) were designed by using the results of virtual screening. Molecular docking indicated that residues Asp86, Glu81, Leu83, Lys89, Lys33, and Gln131 formed hydrogen bonds with the ligand, which affected activity of the ligand. Based on the QSAR model prediction and molecular docking, two candidate compounds, **I13** and **I60** (predicted pIC_50_ > 8, docking score > 10), with the most potential research value were further screened out. MD simulations of the corresponding complexes of these two candidate compounds further verified their stability. This study provided valuable information for the development of new potential CDK2 inhibitors.

## 1. Introduction

Cancer is a serious threat to human health and the sustained cellular proliferation has been considered a key hallmark of cancer [1]. The proliferation of mammalian cells is controlled by the cell cycle in which cyclin-dependent kinases (CDKs) regulate the critical phases [2,3]. CDKs are a group of enzymes that directly regulate the orderly completion of the cell cycle [4]. The human genome encodes 21 CDKs, which typically need to associate with the corresponding cyclins to be active [5]. Previous studies suggested that the inhibition of CDKs could play a crucial role in restraining cancer and frequent dysregulation of CDKs in cancer cells has made CDKs as remarkable targets for cancer therapy [6,7].

**Figure 1:**
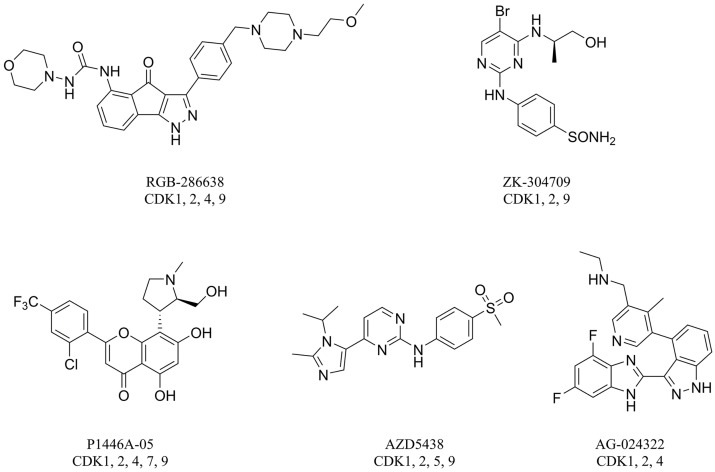
Structures of RGB-286638, ZK-304709, P1446A-05, AZD5438, and AG-024322.

CDK2 that intervenes in the cell cycle at the G1 and S phases is an important member of the CDK family. During the G1 phase, CDK2 paired with cyclin E leads to hyperphosphorylation of the retinoblastoma tumor suppressor protein (Rb), which causes full release of the suppression of the E2F family of transcription factors, which drives cells into the G1/S transition. During the S phase, CDK2 binds to cyclin A to promote the phosphorylation and inactivation of E2F, which results in S phase progression [8]. CDK2 is considered a significant therapeutic target for cancer therapy because it plays a vital role in regulating the cell cycle [7]. To date, numerous CDK2 inhibitors have been designed and developed as potential cancer therapeutic agents such as RGB-286638, ZK-304709, P1446A-05, AZD5438, and AG-024322 (Figure 1). These CDK2 inhibitors lacked selectivity within the CDK family and also inhibited many other kinases. These off-target kinase interactions and the non-selective inhibition of CDKs have detrimental effects on normal cells and result in generalized cytotoxicity with concomitant undesirable adverse effects in clinical trials. Hence, clinical trials of these inhibitors were discontinued because of disadvantageous pharmacological properties [2,3,8].

**Figure 2:**
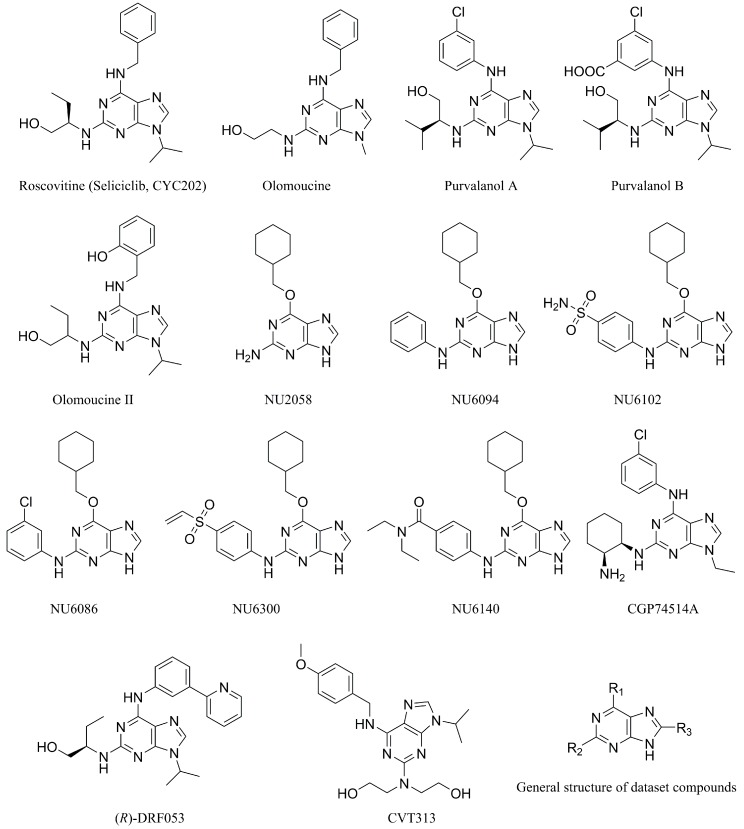
The structures of some CDK inhibitors have the purine scaffold and the general structure of the compounds studied here.

There are many CDK inhibitors having the purine scaffold such as Roscovitine, Olomoucine, Purvalanol A, Purvalanol B, Olomoucine II, NU2058, NU6094, NU6102, NU6086, NU6300, NU6140, CGP74514A, (*R*)-DRF053, and CVT313 (Figure 2) [3,7,9,10,11]. Purine heterocycles have caught particularly extensive attention as the most regularly studied scaffolds for the development of CDK2 inhibitors. Recently, a series of purine derivatives have been reported as CDK2 inhibitors with specificity for CDK2 over other CDKs [12,13]. To further explore the mechanism of action and structure–activity relationship (SAR) and to rapidly design new candidate compounds as potential CDK2 inhibitors at low cost and high efficiency, computer-aided drug design (CADD) was utilized to perform a systematic study on this series of inhibitors. The general structure of the compounds studied here is shown in Figure 2. Three-dimensional quantitative SAR (3D-QSAR) methods including comparative molecular field analysis (CoMFA) [14], comparative molecular similarity indices analysis (CoMSIA) [15], and Topomer CoMFA [16] were used to elucidate the SAR of this series of inhibitors. Topomer Search [17] was applied to perform R-group virtual screening and then the results were used to design novel candidate compounds as potential CDK2 inhibitors. Molecular docking was used to explore the binding mode between the inhibitors and receptor as well as the binding mode between the newly designed candidate compounds and receptor. The QSAR model prediction and molecular docking results were considered together to identify candidate compounds as having the most potential research value. Molecular dynamics (MD) simulations were implemented on the corresponding complexes of the identified candidate compounds to verify their stability and obtain detailed information about the mechanism of action between the ligand and the receptor. This study could provide important references for the synthesis and design of new potential CDK2 inhibitors.

## 2. Results and Discussion

### 2.1. Validation of 3D-QSAR Models

Before a QSAR model can be explored, it must first be validated internally and externally [18]. As can be seen from Table S1, the internal validation parameter q^2^ value of most models is greater than 0.500, which indicated that these models are acceptable. To select better CoMFA and CoMSIA models in terms of bigger q^2^, bigger r^2^, smaller standard error of estimate (SEE), and bigger *F* values, the non-linear, multi-objective scoring technique Pareto ranking, which is widely used in engineering, was utilized [19]. As a result, the CoMFA and CoMSIA models with different patterns in internal and external predictivity were selected. In order to further identify CoMFA and CoMSIA models with the best predictivity among these comparable models, $r_{m}^{2}$_(overall)_ and ${r^{\prime }}_{m}^{2}$_(overall)_ metrics of these models were calculated and compared. As can be seen from Table 1, the $r_{m}^{2}$_(overall)_ (0.866) and ${r^{\prime }}_{m}^{2}$_(overall)_ (0.865) of the CoMFA model based on the alignment 1 are similar to the $r_{m}^{2}$_(overall)_ (0.876) and ${r^{\prime }}_{m}^{2}$_(overall)_ (0.875) of the CoMFA model based on alignment 2. However, the $r_{m}^{2}$_(test)_ (0.902) and ${r^{\prime }}_{m}^{2}$_(test)_ (0.901) of the CoMFA model based on alignment 1 are much higher than the $r_{m}^{2}$_(test)_ (0.867) and ${r^{\prime }}_{m}^{2}$_(test)_ (0.866) of the CoMFA model based on alignment 2, which indicates the former’s external predictive ability better. In terms of the $r_{m}^{2}$_(overall)_ and ${r^{\prime }}_{m}^{2}$_(overall)_ values, the CoMSIA (HSE) model based on the alignment 1 and the CoMSIA (DHSE) model based on the alignment 2 are the top two CoMSIA models. The $r_{m}^{2}$_(overall)_ (0.857) and ${r^{\prime }}_{m}^{2}$_(overall)_ (0.855) for the CoMSIA (HSE) model based on the alignment 1 are comparable to the $r_{m}^{2}$_(overall)_ (0.850) and ${r^{\prime }}_{m}^{2}$_(overall)_ (0.849) of the CoMSIA (DHSE) model based on alignment 2. However, the $r_{m}^{2}$_(test)_ (0.897) and ${r^{\prime }}_{m}^{2}$_(test)_ (0.891) for the CoMSIA (HSE) model based on the alignment 1 are much lower than the $r_{m}^{2}$_(test)_ (0.949) and ${r^{\prime }}_{m}^{2}$_(test)_ (0.945) for the CoMSIA (DHSE) model based on alignment 2, which indicates that the latter’s external predictive power is better. Considering their suitable $r_{m}^{2}$_(overall)_ metrics and high $r_{m}^{2}$_(test)_ metrics values, the CoMFA model based on the alignment 1 and the CoMSIA (DHSE) model based on alignment 2 were regarded as the optimal CoMFA and CoMSIA model for further analysis and external validation, respectively.

The optimal CoMFA model obtained progressive scrambling Q^2^ of 0.397 and the optimal CoMSIA model obtained progressive scrambling Q^2^ of 0.596, respectively. Progressive scrambling slopes (*dq^2^′/dr^2^_yy_’*) for the optimal CoMFA model and the optimal CoMSIA model are 1.159 and 1.129, respectively. The progressive scrambling Q^2^ of the two models are all greater than 0.350 and the progressive scrambling slopes of the two models are all less than 1.200 and near unity, which signifies that the models are robust and stable [20,21]. The test set not used to construct the 3D-QSAR models was used to evaluate the reliability and predictive ability of the obtained models. Various external validation statistical parameters were calculated (Table 2). The $r_{\mathrm{pred}}^{2}$ (q^2^_ext_) values of the optimal CoMFA, CoMSIA, and Topomer CoMFA models are 0.991, 0.990, and 0.962, respectively, which indicated that these models have good predictive power. For the optimal CoMFA model: q^2^ = 0.743 > 0.500, $R_{\mathrm{test}}^{2}$ = 0.991 > 0.600, [($R_{\mathrm{test}}^{2}$ − $R_{0}^{2}$)/$R_{\mathrm{test}}^{2}$] = −0.008 < 0.100, [($R_{\mathrm{test}}^{2}$ − ${R^{\prime }}_{0}^{2}$)/$R_{\mathrm{test}}^{2}$] = −0.008 < 0.100, and the corresponding 0.850 ≤ k = 0.994 ≤ 1.150, 0.850 ≤ k’ = 1.006 ≤ 1.150; for the optimal CoMSIA model: q^2^ = 0.808 > 0.500, $R_{\mathrm{test}}^{2}$ = 0.994 > 0.600, [($R_{\mathrm{test}}^{2}$ − $R_{0}^{2}$)/$R_{\mathrm{test}}^{2}$] = −0.002 < 0.100, [($R_{\mathrm{test}}^{2}$ − ${R^{\prime }}_{0}^{2}$)/$R_{\mathrm{test}}^{2}$] = −0.003 < 0.100, and the corresponding 0.850 ≤ k = 0.987 ≤ 1.150, 0.850 ≤ k’ = 1.013 ≤ 1.150; for the Topomer CoMFA model: q^2^ = 0.779 > 0.500, $R_{\mathrm{test}}^{2}$ = 0.971 > 0.600, [($R_{\mathrm{test}}^{2}$ − $R_{0}^{2}$)/$R_{\mathrm{test}}^{2}$] = −0.022 < 0.100, [($R_{\mathrm{test}}^{2}$ − ${R^{\prime }}_{0}^{2}$)/$R_{\mathrm{test}}^{2}$] = −0.022 < 0.100, and the corresponding 0.850 ≤ k = 0.980 ≤ 1.150, 0.850 ≤ k’ = 1.019 ≤ 1.150. These statistics further corroborated the reliable predictive capability of the optimal CoMFA, CoMSIA, and Topomer CoMFA models. From Table 2, it is observed that the optimal CoMFA, CoMSIA, and Topomer CoMFA models established meet the mean absolute error (MAE) based criteria [22]. For the optimal CoMFA model: MAE_(test)_ = 0.127 ≤ 0.1 × training set range (5.903) = 0.590 and MAE_(test)_ + 3 × σ_(test)_ = 0.289 ≤ 0.2 × training set range = 1.181, MAE_(train)_ = 0.151 ≤ 0.1 × training set range = 0.590 and MAE_(train)_ + 3 × σ_(train)_ = 0.514 ≤ 0.2 × training set range = 1.181. For the optimal CoMSIA model: MAE_(test)_ = 0.101 ≤ 0.1 × training set range = 0.590 and MAE_(test)_ + 3 × σ_(test)_ = 0.416 ≤ 0.2 × training set range = 1.181, MAE_(train)_ = 0.154 ≤ 0.1 × training set range = 0.590 and MAE_(train)_ + 3 × σ_(train)_ = 0.619 ≤ 0.2 × training set range = 1.181. For the Topomer CoMFA model: MAE_(test)_ = 0.258 ≤ 0.1 × training set range = 0.590 and MAE_(test)_ + 3 × σ_(test)_ = 0.597 ≤ 0.2 × training set range = 1.181, MAE_(train)_ = 0.295 ≤ 0.1 × training set range = 0.590 and MAE_(train)_ + 3 × σ_(train)_ = 0.982 ≤ 0.2 × training set range = 1.181. The σ_(test)_ and σ_(train)_ denote the standard deviation of the absolute error values for the test set predictions and the standard deviation of the absolute error values for the training set predictions, respectively. In addition, the $r_{m}^{2}$_(test)_, ${r^{\prime }}_{m}^{2}$_(test)_, $r_{m}^{2}$_(avg)_, and ∆$r_{m}^{2}$_(test)_ of the optimal CoMFA model are 0.902, 0.901, 0.902, and 0.001, respectively. The $r_{m}^{2}$_(test)_, ${r^{\prime }}_{m}^{2}$_(test)_, $r_{m}^{2}$_(avg)_, and ∆$r_{m}^{2}$_(test)_ of the optimal CoMSIA model are 0.949, 0.945, 0.947, and 0.004, respectively. The $r_{m}^{2}$_(test)_, ${r^{\prime }}_{m}^{2}$_(test)_, $r_{m}^{2}$_(avg)_, and ∆$r_{m}^{2}$_(test)_ of the Topomer CoMFA mode are 0.831, 0.830, 0.831 and 0.001, respectively. In terms of their validation statistics values, the optimal CoMFA, optimal CoMSIA, and Topomer CoMFA models established are reliable and have good internal and external predictive capabilities, which could be used to accurately predict the activities of novel compounds similar to the compounds in the training set. Therefore, these models were chosen as the final models for subsequent analysis.

### 2.2. 3D-QSAR Statistical Analysis

**Figure 3:**
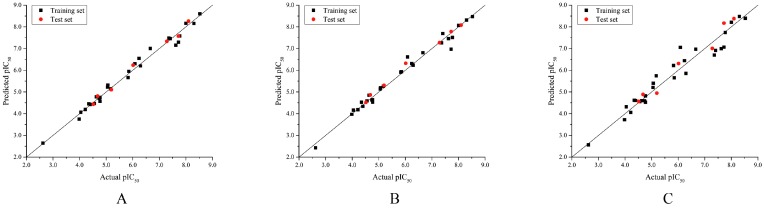
Plot of actual pIC_50_ against predicted pIC_50_ by the optimal CoMFA (**A**), CoMSIA (**B**), and Topomer CoMFA (**C**) models.

As shown in Table S1, the optimal CoMFA model showed cross-validated q^2^ of 0.743, non-cross-validation r^2^ of 0.984, SEE of 0.219 and *F* value of 273.426 with ONC of five. The contributions of the steric fields and electrostatic fields are 0.577 and 0.423, respectively. For the optimal CoMSIA model, it owned cross-validated q^2^ of 0.808, non-cross-validation r^2^ of 0.980, SEE of 0.246 and *F* value of 214.108 with ONC of five. The contributions of steric, electrostatic, hydrogen bond donor, and hydrophobic fields were 0.164, 0.280, 0.221 and 0.335, respectively. The Topomer CoMFA model showed cross-validated q^2^ of 0.779, non-cross-validation r^2^ of 0.941, SEE of 0.412 and *F* value of 91.934 with ONC of four. The predicted pIC_50_ values of the dataset compounds are shown in Table 3. All the residuals between actual and predicted pIC_50_ are less than one logarithm unit, which indicates good predictive performance of the three models. The correlation plot of the actual pIC_50_ against the predicted pIC_50_ for the optimal CoMFA, CoMSIA, and Topomer CoMFA models is illustrated in Figure 3 where all points uniformly distributed around the regression line *Y* = *X*, which suggests the excellent predictive ability and accuracy of the models.

### 2.3. 3D-QSAR Model Contour Map Analysis

In this study, the most potent compound **9** was selected as the reference molecule to demonstrate the contour maps of the optimal CoMFA, CoMSIA, and Topomer CoMFA models using the StDev Coeff field type. For all of the maps contoured by field contribution, the favorable and unfavorable regions of each field type were shown in 80% and 20% contributions, respectively. The contour maps provided clues for the structural modifications required to design new compounds with improved activity.

**Figure 4:**
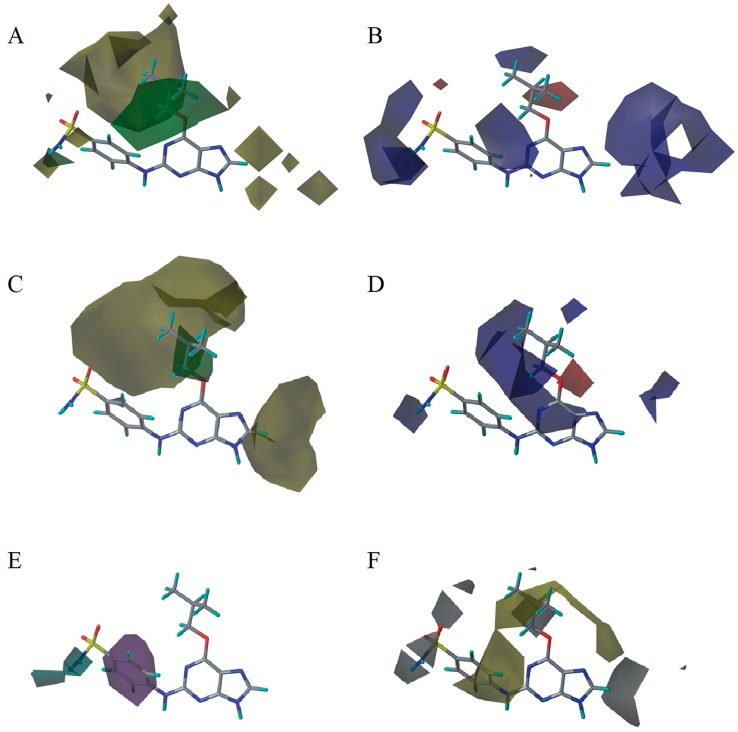
Contour maps of the optimal CoMFA and CoMSIA models. (**A**) CoMFA steric contour. (**B**) CoMFA electrostatic contour. (**C**) CoMSIA steric contour. (**D**) CoMSIA electrostatic contour. (**E**) CoMSIA hydrogen bond donor contour. (**F**) CoMSIA hydrophobic contour. Steric bulk favored areas that are in green and unfavorable areas in yellow (**A**,**C**). Electropositive favored areas are in blue and electronegative favored areas are in red (**B**,**D**). The hydrogen bond donor favored areas that are in cyan and unfavorable areas that are in purple (**E**). Hydrophobic favored areas are in green and unfavorable areas are in white (**F**).

In the CoMFA steric contour map (Figure 4A), the region of the R_1_ substituent was flanked by a medium-sized green contour within which steric bulk is favored and a big yellow contour where occupation is disfavored. For instance, compounds **1**, **2,** and **3** with a cyclohexyl ring in the green area showed higher activities than compounds **4**, **5,** and **6** with –H as substituent R_1_ at this position, respectively. Likewise, compounds **16**, **13,** and **6** have an order of potencies of **16** (ethyl) > **13** (ethynyl) > **6** (–H). In general, appropriately increasing the substituent volume in this region is favorable to the activity but adding an excessively large substituent at this position adversely affects the activity. The trend can be observed in the order of the activity for compound **9** (2-methyl-1-propoxy) > **7** (*n*-propoxy) ≈ **8** (*i*-propoxy) > **11** (triisopropylsilylethynyl). The substituents of compounds **9**, **7,** and **8** in the R_1_ are close to the green region, which favors the inhibitory activity. However, as the volume of substituent there further increased, the substituent of compound **11** fell into the region occupied by yellow contours and did not favor the activity. Compound **11** with triisopropylsilylethynyl as substituent R_1_ showed better activity than compound **13** with ethynyl as substituent R_1_ because the linear ethynyl group sat in an open region in the steric contour where steric bulk is neither favored or disfavored. A small green contour near the –NH_2_ moiety of sulfonamide group of reference molecule at the R_2_ position indicated that bulky substitution in this region might enhance the activity. For example, compound **3** (4-sulfamoylanilino) > **2** (anilino), **6** (4-sulfamoylanilino) > **5** (anilino), and **13** (4-sulfamoylanilino) > **12** (anilino). Some medium-sized yellow contours around the R_3_ substitution position suggests that bulky substitution in this region will reduce the activity. In addition to compound **34** and **35**, most of the compounds with substituents introduced at the R_3_ position have lower activity than compound **1** (–H).

The electrostatic contour map of the CoMFA is displayed in Figure 4B. The area where the R_1_ substituent is located is surrounded by medium-sized blue and red contours, which indicates that introducing more electronegative groups or atoms on the red region will improve the activity while more electropositive groups or atoms near the blue area will increase the activity. For example, compound **1** has a cyclohexylmethoxy substituent in this region. The oxygen atom of the substituent was directed toward red contour and the electron donating group cyclohexylmethyl portion, which is relatively electropositive near the blue region. Hence, compound **1** is more potent than **4** (–H). Similarly, compound **3** (cyclohexylmethoxy) > **6** (–H), compounds **9** (2-methyl-1-propoxy), **3** (cyclohexylmethoxy), **7** (*n*-propoxy), **8** (*i*-propoxy), **11** (triisopropylsilylethynyl) and **10** (prop-2-ynyloxy) are generally more active than compounds **18** (phenyl), **19** (3-methoxyphenyl), **21** (3-phenylphenyl), **20** (4-methoxyphenyl), **16** (ethyl), **22** (benzo[d][1,3]dioxol-5-yl), and **23** (4-dibenzofuryl). As shown in Figure 4B, there is a medium-sized blue contour at R_2_, which indicated that adding positively charged substituents in this area can improve activity while substituents that are negatively charged can reduce the activity. A large blue contour occupying the R_3_ position implied that introducing positively charged substituents therein could enhance the activity of compounds. For this reason, compounds **29** (2-chlorophenyl), **30** (2-methylphenyl), and **32** (2-methylphenol) are more potent than compounds **27** (*i*-propyl), **26** (ethyl), **25** (methyl), **28** (phenyl), and **31** (trifluoromethyl). The activities of these compounds decrease as the negative charges on their substituent increase.

As shown in Figure 4C,D, the optimal CoMSIA model tends to have more sharp steric and electrostatic contour maps but is still basically similar to those of the optimal CoMFA model. CoMSIA hydrogen bond donor contours are shown in Figure 4E. The cyan contour indicated a favorable hydrogen bond donor substituent region while a purple contour indicated a region that is unfavorable for hydrogen bond donor groups. Cyan contours near the –NH_2_ moiety of the sulfonamide of the R_2_ substituent indicated a hydrogen bond donor introduced there, which can enhance the activity of compounds. This scenario can be observed by the order of the activity for compound **3** (4-sulfamoylanilino) > **2** (anilino) > **1** (amino), **6** (4-sulfamoylanilino) > **5** (anilino) > **4** (amino).

In the hydrophobic contour map of the optimal CoMSIA model (Figure 4F), the presence of white contours observed near the substituent R_1_ position indicates that the hydrophilic substituent is favorable for activity. Meanwhile, a fairly large, yellow contour crossed the R_1_, R_2_, and R_3_ regions within which hydrophobic substituents are favored. This can be illustrated by the fact that compound **2** (cyclohexylmethoxy) > **14** (prop-1-ynyl) > **15** (phenylethynyl) > **12** (ethynyl) > **5** (–H). The large yellow contour around the R_2_ substitution implied that hydrophobic group would increase the activity of the compound. On the other hand, around the sulfonamide group of the reference molecule, some scattered white areas are regions where more hydrophilic substituents are desirable for the improvement of the activity. In the R_3_ substituent area, there is a medium-sized white contour that indicated more hydrophilic substituents are preferred to produce higher inhibitory activity. This conclusion is consistent with the experimental results that, in the R_3_ position, most of the less active compounds all possess a hydrophobic group near the white contour, which has a detrimental effect on the activity such as compound **35**, **34**, **29**, **30**, **32**, **27**, **26**, **33**, **25**, **28**, and **31**. In addition, R_3_ substituents of **25** and **31** fell into the white area where the hydrophobic substituent is not favored. Therefore, **25** (methyl) > **31** (trifluoromethyl). As can be seen from Figure S1, the steric and the electrostatic contour maps of the Topomer CoMFA model are similar to those of the optimal CoMFA and CoMSIA models.

### 2.4. Virtual Screening Results and Molecular Design

In this study, using Topomer Search, 1694 R_1_ hit fragments and 28 R_2_ hit fragments were obtained, which have higher predicted R-group contribution to activity than corresponding R_1_ or R_2_ groups of the training set. In general, the hit R-group structures have high predicted activity and are also similar to the corresponding training set R-group structures, which were selected to replace the R-groups of the most potent compound in the training set [23]. Therefore, all hit R-group fragments were filtered by compound **9**. Finally, 76 R_1_ hits and nine R_2_ hits were selected from the R-group virtual screening results, respectively. The R_1_ and R_2_ groups of compound **9** were alternately replaced by the corresponding most active R_1_ and R_2_ groups in the training set and the corresponding selected 76 R_1_ and nine R_2_ hit fragments. As a result, 769 compounds were designed. These compounds were constructed by using the procedures applied to construct the dataset compounds and the optimal CoMFA, CoMSIA, and Topomer CoMFA models predicted their activities. Among the 769 compounds, 31 newly designed compounds with predicted activities of >8, which are comparable with the most potent compound **9**. However, their selectivity towards other CDKs or similar kinases is unknown, which means they may have some side-effects. The structures and predicted activities of the 31 novel candidate compounds are presented in Table S2.

### 2.5. Docking Analysis

**Figure 5:**
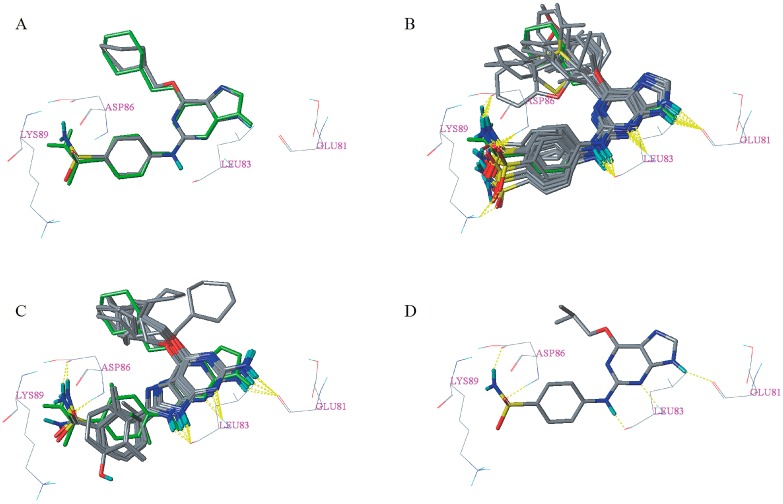
Re-docking result of the cognate ligand (**A**) and docking results of dataset compounds (**B**,**C**) and compound **9** (**D**). The cognate ligand was displayed in the green stick model. Hydrogen bonds were shown as yellow dashed lines and non-polar hydrogens were removed for clarity.

To get insights into the binding mode between the ligand and receptor, all dataset compounds and the 31 newly designed candidate compounds were docked into the active site of the receptor. First, to validate the accuracy of the docking and the rationality of parameters utilized in docking, the cognate ligand was docked into the active site of the protein receptor by the re-docking method. As can be seen from Figure 5A, the co-crystallized conformation and the re-docked conformation of the cognate ligand superimposed very well with each other in the same binding site. Moreover, the RMSD value between the co-crystallized conformation and the re-docked conformation of the cognate ligand is 0.850 Å, which indicates the rationality and reliability of the docking [24].

Docking results of the dataset compounds are listed in Table S3. All of the dataset compounds have a total score greater than four, which signifies that they can be considered to be specific ligands of the corresponding receptor protein [25]. In addition, Cscore of 27 dataset compounds are greater than or equal to three. According to the docking results, the binding mode types of dataset compounds can be divided into type I (Figure 5B) and type II (Figure 5C). For type I, its binding pattern is nearly identical to that of the cognate ligand. However, for type II, the purine scaffold has flipped 180° compared with the type I binding mode. The less active compounds **25**–**35** adopt type II binding mode for the reason that their R_3_ substitutions are too bulky and in order to avoid unacceptable steric clash, which is consistent with the steric contour maps of 3D-QSAR models in the R_3_ position. This might be one reason why the activity of compounds (such as **9**, **3**, **7**, **8**, **11**, **10**, **17**, **18**, **19**, **21**, **20**) adopting type I binding mode is generally higher than that of compounds adopting type II binding mode. Each of the more active compounds with the type I binding mode made a conserved triplet of hydrogen bonds between 9-H, *N*-3, 2-amino group, and the backbone carbonyl moiety of Glu81 and amide and carbonyl moieties of Leu83, respectively. The sulfonamide group of these compounds formed hydrogen bonds with Asp86 or Lys89, which is consistent with the cyan contour of the optimal CoMSIA model near the sulfonamide group. However, as can be seen from Figure 5C, most of the compounds (such as **25**, **26**, **27**, **28**, and **31**) adopting the type II binding mode only formed the conserved triplet of hydrogen bonds with the residues Glu81 and Leu83, which might be one reason for the difference in potency between the compounds adopting the two different binding mode types. To further illustrate the interaction mechanism between dataset compounds and the corresponding receptor, the most active compound **9** was taken as an example for detailed analysis [26,27]. As shown in Figure 5D, five hydrogen bonds formed between compound **9** and the surrounding residues Glu81, Leu83, and Asp86. The corresponding hydrogen bond distances were measured to be 1.890 Å (Glu81-C=O∙∙∙H–N), 2.190 Å (Leu83-N-H∙∙∙N=C), 1.830 Å (Leu83-C=O∙∙∙H-N), 2.020 Å (Asp86-N-H∙∙∙O=S), and 2.050 Å (Asp86-C=O∙∙∙H-N), respectively.

**Figure 6:**
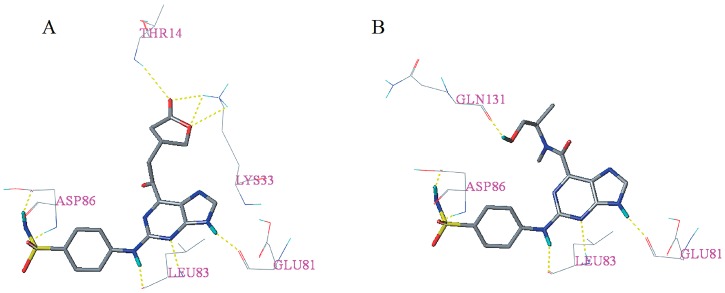
Docking results of candidate compounds **I13** (**A**) and **I60** (**B**). Hydrogen bonds were shown as yellow dashed lines and non-polar hydrogens were removed for clarity.

Docking results of the 31 novel candidate compounds are presented in Table S4. Surflex–Dock total score of each candidate compound is greater than eight. Except **I46**, **I173**, and **I63**, Cscore of the other 29 candidate compounds is greater than or equal to three. In addition, candidate compounds **I13**, **I21**, **I33**, **I40**, **I44**, **I60**, **I155,** and **I190** showed high similarity [28] (>0.800) with the cognate ligand. These results indicated that most of the newly designed candidate compounds may have good binding affinity. As can be seen from Figure 6, candidate compounds **I13** and **I60** having a total score of >10, the Cscore equal to five and have a similarity of >0.800 formed hydrogen bonds with residues Glu81, Leu83, Asp86, Lys33, Thr14, and Gln131, respectively. The corresponding hydrogen bond distances between **I13** and receptor are 1.850 Å (Glu81-C=O∙∙∙H-N), 2.330 Å (Leu83-N-H∙∙∙N=C), 1.990 Å (Leu83-C=O∙∙∙H-N), 1.940 Å (Asp86-N-H∙∙∙O=S), 2.040 Å (Asp86-C=O∙∙∙H-N), 2.690 Å (Thr14-N-H∙∙∙O=C), 2.010 Å (Lys33-N-H∙∙∙O=C), 2.480 Å (Lys33-N-H∙∙∙O-C), 2.750 Å (Lys33-N-H∙∙∙O-C). On the other hand, the hydrogen bond distances between **I60** and receptor are 1.850 Å (Glu81-C=O∙∙∙H-N), 2.490 Å (Leu83-N-H∙∙∙N=C), 2.170 Å (Leu83-C=O∙∙∙H-N), 1.870 Å (Asp86-N-H∙∙∙O=S), 2.010 Å (Asp86-C=O∙∙∙H-N), 1.850 Å (Gln131-C=O∙∙∙H-O).

### 2.6. MD Simulations Analysis

In this scenario, in order to further explore the possible interaction mechanisms between 1H1S and two candidate compounds **I13** and **I60** (predicted pIC_50_ > 8, docking score > 10), with the main aim at exploring the conformational changes of the ligands and the receptor occurring in each docked complex. Ten ns MD simulations were executed on two docked complex structures **1H1S-I13** and **1H1S-I60**, respectively.

**Figure 7:**
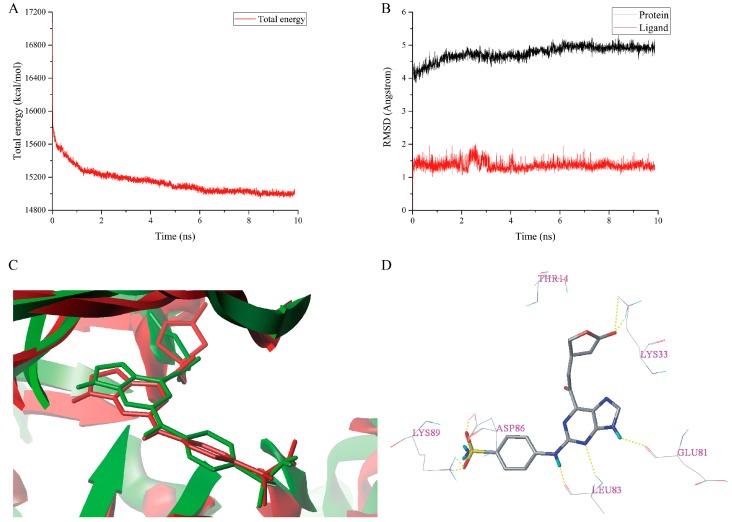
MD simulations result of the complex **1H1S-I13**. (**A**) The total energy plot. (**B**) The RMSDs plot. (**C**) The superimposition of the initial docked structure (red) of complex **1H1S-I13** and the average structure (green) of MD simulation of complex **1H1S-I13**. (**D**) The conformation of **I13** after MD simulations. Hydrogen bonds were shown as yellow dashed lines and non-polar hydrogens were removed for clarity.

The total energy plot and the RMSDs plot for the docked complex **1H1S-I13** are shown in Figure 7A,B, respectively. The MD simulations results revealed that the total energy was fluctuated around 15,100 kcal/mol and the protein backbone RMSD tended to be stable and fluctuated around 4.600 Å after 6 ns simulations and the RMSD of the ligand stabilized around 1.500 Å after 5 ns simulations, which suggests that the structure of the complex had reached a converged state. The superimposition of the initial docked structure of complex **1H1S-I13** with the average structure obtained from the last two ns MD simulations of the complex is shown in Figure 7C. The conformation and orientation of the **I13** are similar before and after MD simulations, which indicates that the docking result is reliable. As can be seen from Figure 6A and Figure 7D, the number and the length of the hydrogen bonds between **I13** and the receptor have changed after MD simulations. For instance, one hydrogen bond arising from Thr14 and **I13** and the two hydrogen bonds between sulfonamide group of the **I13** and residue Asp86 have disappeared. Before MD simulations, there were three hydrogen bonds between **I13** and Lys33 and the corresponding distances were 2.010 Å (Lys33-N-H∙∙∙O=C), 2.480 Å (Lys33-N-H∙∙∙O-C), and 2.750 Å (Lys33-N-H∙∙∙O-C). However, after MD simulations, **I13** only formed two hydrogen bonds with Lys33. The distances of the two hydrogen bonds are 1.480 Å (Lys33-N-H∙∙∙O=C) and 2.760 Å (Lys33-N-H∙∙∙O=C). Three new hydrogen bonds are formed between **I13** and the receptor. Their distances are 1.520 Å (Asp86-O-H∙∙∙O=S), 1.460 Å (Lys89-N-H∙∙∙O=S), and 2.760 Å (Lys89-N-H∙∙∙O=S). The hydrogen bond distance between the 9-H and Glu81, the *N*-3 and Leu83, and the 2-NH and Leu83 was changed from 1.850 to 1.890 Å, 2.330 to 2.440 Å, and 1.990 to 1.790 Å after MD simulations.

**Figure 8:**
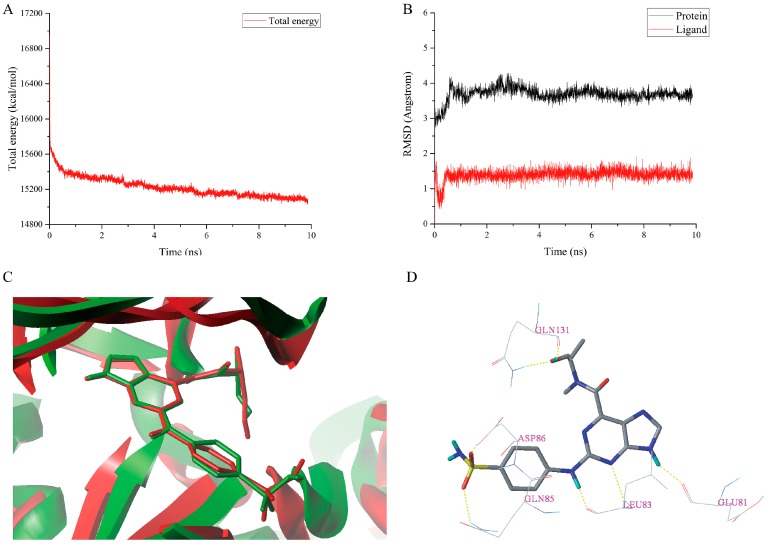
MD simulations result of the complex **1H1S-I60**. (**A**) The total energy plot. (**B**) The RMSDs plot. (**C**) The superimposition of the initial docked structure (red) of complex **1H1S-I60** and the average structure (green) of MD simulation of complex **1H1S-I60**. (**D**) The conformation of **I60** after MD simulations. Hydrogen bonds were shown as yellow dashed lines and non-polar hydrogens were removed for clarity.

The total energy plot and the RMSDs plot for the docked complex **1H1S-I60** are shown in Figure 8A,B, respectively. The MD simulations results revealed that the total energy was fluctuated around 15,200 kcal/mol after 5 ns. The protein backbone RMSD tended to be stable and fluctuated around 3.600 Å after 4 ns simulations. The RMSD of the ligand stabilized around 1.400 Å after 1 ns simulations, which signified that the complex **1H1S-I60** had reached equilibrium. The superimposition of the initial docked structure of complex **1H1S-I60** with the average structure obtained from the last 2 ns MD simulations of the complex is shown in Figure 8C. The conformation of the **I60** is stable without significant changes after MD simulations, which indicates the rationality and validity of the docking result. From Figure 6B and Figure 8D, it could be observed that the two hydrogen bonds between the sulfonamide group of the **I60** and residue Asp86 have disappeared after MD simulations. However, three new hydrogen bonds are formed between **I60** and Asp86, Gln85 and Gln131 at the distance of 1.380 Å (Asp86-O-H∙∙∙O=S), 2.750 Å (Gln85-N-H∙∙∙O=S), and 2.320 Å (Gln131-N-H∙∙∙O-C), respectively. The hydrogen bond distance between the 9-H and Glu81, the *N*-3 and Leu83, the 2-NH and Leu83, and the –OH and Gln131 was changed from 1.850 to 1.790 Å, 2.490 to 2.370 Å, 2.170 to 1.740 Å, and 1.850 to 2.890 Å after MD simulations.

## 3. Materials and Methods

All the calculations in this study were performed by using the commercially available SYBYL-X 2.0 software package (Tripos Inc., St. Louis, MO, USA).

### 3.1. Dataset

In this study, 35 CDK2 inhibitors were collected from the literature as dataset compounds, which include a diverse subset of the structures and contain a wide variety of functional groups [12,13]. The experimental IC_50_ values were converted into pIC_50_ (−log IC_50_) values, which were used as dependent variables in the QSAR models. The chemical structures of all compounds along with their pIC_50_ values are shown in Table 4. The pIC_50_ values of the dataset compounds uniformly distribute over the entire pIC_50_ value range (2.620–8.523), which is fit for 3D-QSAR studies. The total dataset compounds were divided into a training set and a test set in 4:1 ratio. The training set contains structurally diverse compounds representing all those chemical classes in the whole dataset and the activities of the training set compounds span the entire pIC_50_ value range of the dataset, which can help the QSAR models portray more accurately what structural modifications are favorable or detrimental to activity. The test set compounds were selected randomly, but it is ensured that they represent the whole range of both structure diversity and activity of the training set compounds [29]. The training set was used to build 3D-QSAR models and the test set was used to evaluate and validate the predictive quality and reliability of the 3D-QSAR models obtained.

### 3.2. Structure Preparation

All the compound structures were constructed by using the SYBYL’s Sketcher and their atom types and bond types were checked. At first, the most potent compound **9** in the training set was sketched and then its geometry was optimized through MAXIMIN2 of SYBYL. Using the Tripos force field [30], energy minimization was performed by the Powell method [31] with a gradient termination criterion of 0.005 kcal/(mol∙Å) and a maximum iteration of 10,000. Partial atomic charges were assigned by Gasteiger-Hückel charges [32] and all other parameters were set as the default. The optimized structure of compound **9** was selected as the template structure to sketch all of the rest of compounds, which later also underwent an energy minimization procedure [27].

### 3.3. Molecular Docking

For investigating interactions and the binding mode between the ligand compounds and target receptor, all the compounds were docked into the active site of the X-ray crystallographic structure of the target receptor (PDB ID: 1H1S) by using the Surflex–Dock method [33]. Before docking, both the ligand and the receptor protein were prepared [26]. The co-crystallized ligand was extracted from the active site. Water was removed from 1H1S. The backbone and the sidechain of the protein were repaired. Termini were set to be charged. Hydrogens were added to the ligand and receptor. The protonation type of the residues that are within 6 Å of cognate ligand and that may have more than one protonation state at the near neutral pH, which was set to favor hydrogen bonding. AMBER7 FF99 atom types were assigned to the receptor and ligand. Gasteiger-Hückel charges were calculated for the ligand while AMBER7 FF99 charges were computed for the receptor. The sidechain amides in all Asn and Gln residues were oriented in the direction of maximizing potential hydrogen bonding. Lastly, a staged minimization using default setting except for the force field option set as AMBER7 FF99 was conducted to optimize the protein-ligand complex. With the default parameters, the protomol was generated by the ligand-mode [34]. First, with the docking mode set to Surflex–Dock Geomx (SFXC), the extracted cognate ligand was docked back into the active site to check the rationality of parameters utilized in docking by comparing the conformations and the root mean square deviation (RMSD) value between the original orientation and the re-docked orientation of the co-crystallized ligand. Then, all dataset compounds and newly designed candidate compounds were docked into the active site of the receptor.

### 3.4. Alignment

One of the most significant factors affecting the predictive ability and the statistical parameters quality of a 3D-QSAR model is the molecular alignment adopted by compounds [34,35]. Many methods have been proposed for aligning compounds in preparation for QSAR, which basically falls into three categories: substructure overlap, pharmacophore overlap, and docking [36]. In this study, three different alignment methods were tested to obtain better QSAR models.

**Figure 9:**
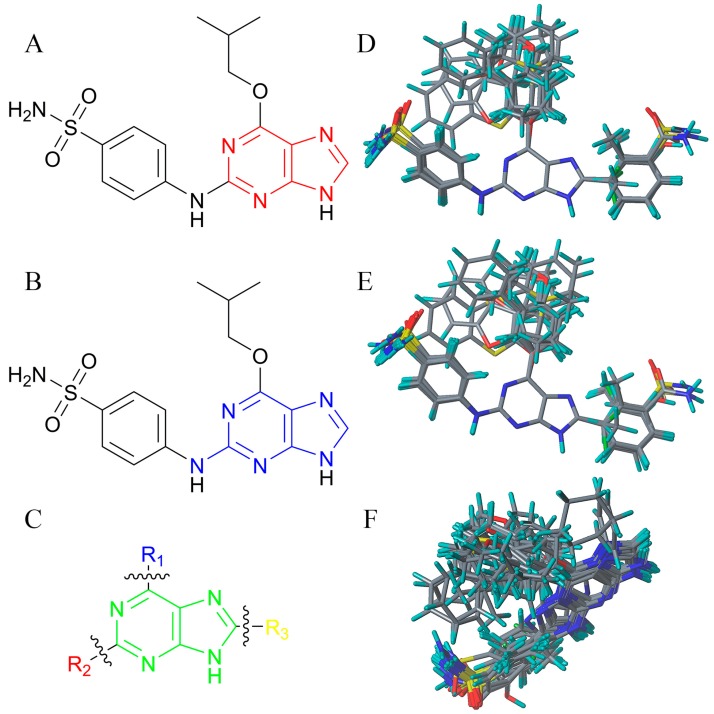
The template of alignment and graph of molecular alignment. (**A**) The common core used in alignment 1. (**B**) The MCS used in alignment 2. (**C**) The R-groups fragmentation scheme. (**D**) Aligned molecules based on alignment 1. (**E**) Aligned molecules based on alignment 2. (**F**) Aligned molecules based on alignment 3.

Alignment 1 is the database alignment, which belongs to substructure overlap. This is a rigid fitting of the common core of the molecules to a template. The most potent compound **9** was chosen as the template. The common core is shown in Figure 9A and the aligned molecules are shown in Figure 9D. Alignment 2 is distill rigid alignment, which is a variant of the substructure overlap. A rigid alignment attempts to align molecules in a database to a template molecule on a maximum common substructure (MCS) produced by distill. The torsion angles of the rotatable bonds in the MCS for each molecule being aligned were adjusted to match those of the template [27]. The most active compound **9** was selected as template. All other settings were the default. The MCS found is shown in Figure 9B and the aligned molecules are shown in Figure 9E. Alignment 3 is docking-based alignment. This strategy has the nice advantage of setting the sidechain conformation and the positions of the pharmacophore elements [37]. All the compounds in the dataset were docked into the receptor’s binding site by using Surflex–Dock. The top-scoring pose for each compound was chosen as the alignment conformation for a subsequent QSAR study. The aligned molecules are shown in Figure 9F.

### 3.5. Creation of CoMFA and CoMSIA Models

All parameters used in CoMFA and CoMSIA studies were defaulted except for the explained. CoMFA is a method involving the shapes of molecules. The concept underlying CoMFA is that differences in the activities of ligands are related to differences in molecular properties represented by molecular fields. The magnitudes of the Tripos standard steric (Lennard-Jones) and electrostatic (Coulombic) fields were determined by the interaction between molecules aligned and a probe atom with a van der Waal radius of sp^3^ carbon and a charge of +1 at regular intervals throughout a defined region [14]. In this study, the defined region extended at least four Å beyond every molecule in *X*, *Y*, and *Z* axes directions and have a two Å interval. The steric and electrostatic fields cutoffs were set at 30 kcal/mol [38]. CoMSIA is an extension of the CoMFA methodology. They differ only in the implementation of the fields. In CoMSIA, five different similarity fields covering the major contributions to ligand binding, namely steric (S), electrostatic (E), hydrophobic (H), hydrogen bond donor (D), and hydrogen bond acceptor (A), were calculated [39]. The region used in CoMSIA was the same as that in CoMFA. However, the probe atom used in CoMSIA has a radius of 1 Å, charge of +1, hydrophobicity of +1, hydrogen bonding donor, and acceptor properties of +1. A Gaussian function was used. Thus, no arbitrary cutoffs were required for CoMSIA fields’ calculations. The five CoMSIA fields may not be very independent of each other and such dependencies of the individual fields often decrease the statistical significance of the results. Thus, 31 possible CoMSIA field combinations were considered when constructing CoMSIA models.

### 3.6. Partial Least Squares Analysis

Partial least squares (PLS) is an extension of the multiple regression (MR). It was applied to linearly correlate the variance in CoMSIA and CoMFA fields to variations in pIC_50_ values of compounds [40]. In this study, PLS was performed in two stages including the first with leave-one-out (LOO) cross-validation to obtain the optimal number of components (ONC), which represents the complexity level of a model and corresponds to the highest cross-validated r^2^ (called q^2^) [41,42]. In the second stage, the ONC, which optimally distinguished the signal from the noise and was used to establish the final QSAR model without cross-validation [43]. The scaling option was set as the CoMFA Standard, which gave each individual field the same potential influence on the resulting QSAR. Moreover, to speed up cross-validation calculations for PLS analysis, the sample-distance PLS (SAMPLS) algorithm was utilized [44]. All remaining settings had default parameters.

### 3.7. Creation of Topomer CoMFA Model

Topomer CoMFA—the second generation of CoMFA—automates the creation of QSAR models that can be submitted to Topomer Search as queries for virtual screening to do lead hopping, to identify novel scaffolds, and for optimizing R-groups [16]. The training set and test set used in CoMFA and CoMSIA studies were selected. All the molecules in the dataset were prepared by Topomers protocol to generate structures suitable for use in Topomer CoMFA and Topomer Search. R-group identification for the molecules was the crucial step for Topomer CoMFA since Topomer CoMFA works via 3D structural comparisons within sets of R-group fragments [45]. In this study, all the molecules were broken into one common core and three R-group fragments, which are shown as core (green), R_1_ (blue), R_2_ (red), and R_3_ (yellow) in Figure 9C. The steric and electrostatic field values for the corresponding fragments were calculated. The field values were regarded as the independent variables and the pIC_50_ values served as dependent variables in an automatic PLS analysis, which was utilized to construct the Topomer CoMFA model. The obtained model was assessed by LOO cross-validation and the pIC_50_ values of the test set compounds were predicted by the model [46].

### 3.8. Validation of 3D-QSAR Models

In this paper, several validation metrics were calculated to evaluate the predictive power and reliability of the 3D-QSAR models derived from the training set and the activities of the compounds in the test set were predicted. In addition, progressive scrambling was performed to examine the robustness of the optimal CoMFA and CoMSIA models. The parameters were defaults. For a predictive QSAR model, the value of $r_{\mathrm{pred}}^{2}$ should be greater than 0.500 [47]. The $r_{\mathrm{pred}}^{2}$ value was calculated by Equation (1).

(1) $$r_{\mathrm{pred}}^{2}=\frac{SD-PRESS}{SD}$$

where *SD* is the sum of squared deviations between observed (i.e., actual) activities of the test set compounds and the mean activity of the training set compounds. *PRESS* is the sum of squared deviations between the actual and the predicted activities of the test set compounds. For a QSAR model to have high predictive power, it also should satisfy all of the following conditions: q^2^ > 0.500, $R_{\mathrm{test}}^{2}$ > 0.600, $R_{0}^{2}$ or ${R^{\prime }}_{0}^{2}$ should be close to $R_{\mathrm{test}}^{2}$, i.e., [($R_{\mathrm{test}}^{2}$ − $R_{0}^{2}$)/$R_{\mathrm{test}}^{2}$] < 0.100 or [($R_{\mathrm{test}}^{2}$ − ${R^{\prime }}_{0}^{2}$)/$R_{\mathrm{test}}^{2}$] < 0.100, and the corresponding 0.850 ≤ *k* ≤ 1.150 or 0.850 ≤ *k′* ≤ 1.150 [48]. In addition, a QSAR model with good prediction performance should pass the following MAE-based criteria: *MAE* ≤ 0.1 × training set range and *MAE* + 3 × σ ≤ 0.2 × training set range [22]. In this case, the σ value denotes the standard deviation of the absolute error values for the corresponding set (test set or training set) data. These statistics and a series of $r_{m}^{2}$ metrics [47,49] for external validation were calculated by using the formulas below.

(2) $$R_{\mathrm{test}}^{2}=\frac{{\left[\sum \left(Y_{obs}-\bar{Y_{obs}}\right)\left(Y_{pred}-\bar{Y_{pred}}\right)\right]}^{2}}{\sum {\left(Y_{obs}-\bar{Y_{obs}}\right)}^{2}\sum {\left(Y_{pred}-\bar{Y_{pred}}\right)}^{2}},$$

(3) $$k=\frac{\sum Y_{obs}Y_{pred}}{\sum Y_{pred}^{2}},$$

(4) $$k^{\prime }=\frac{\sum Y_{obs}Y_{pred}}{\sum Y_{obs}^{2}},$$

(5) $$R_{0}^{2}=1-\frac{\sum {\left(Y_{pred}-kY_{pred}\right)}^{2}}{\sum {\left(Y_{pred}-\bar{Y_{pred}}\right)}^{2}},$$

(6) $${R^{\prime }}_{0}^{2}=1-\frac{\sum {\left(Y_{obs}-k^{\prime }Y_{obs}\right)}^{2}}{\sum {\left(Y_{obs}-\bar{Y_{obs}}\right)}^{2}},$$

(7) $$r_{m}^{2}=R_{\mathrm{test}}^{2}\left(1-\sqrt{\left|R_{\mathrm{test}}^{2}-R_{0}^{2}\right|}\right),$$

(8) $${r^{\prime }}_{m}^{2}=R_{\mathrm{test}}^{2}\left(1-\sqrt{\left|R_{\mathrm{test}}^{2}-{R^{\prime }}_{0}^{2}\right|}\right),$$

(9) $$MAE=\frac{\sum \left|Y_{obs}-Y_{pred}\right|}{n},$$

In Equation (2)–(9), *Y_obs_* and *Y_pred_* correspond to the observed (i.e., actual) and predicted activities, respectively, of the test set compounds and n is the number of the corresponding compounds. In this scenario, the *MAE* was calculated for the training set and the test set, respectively. Therefore, the n in Equation (9) is the number of compounds in the corresponding set. $R_{\mathrm{test}}^{2}$ is the correlation coefficient (with intercept) between the observed and predicted activities of the test set compounds, $R_{0}^{2}$ (predicted versus observed activities), and ${R^{\prime }}_{0}^{2}$ (observed versus predicted activities) are the correlation coefficients for regressions (without intercept) through the origin, *k*, and *k’*, which are the slopes of regression lines through the origin corresponding to $R_{0}^{2}$ and ${R^{\prime }}_{0}^{2}$, respectively [48]. Furthermore, the $r_{m}^{2}$ metrics can be applied for the overall dataset by using LOO-predicted activities for the training set and the QSAR model predicted activities for the test set. The resulting $r_{m}^{2}$_(overall)_ metrics could be used for identifying better models from many comparable QSAR models [49]. A QSAR model with good predictive quality should meet the following guidelines: $r_{m}^{2}$_(avg)_, namely the average of $r_{m}^{2}$ and ${r^{\prime }}_{m}^{2}$, and the value of $r_{m}^{2}$ metrics should be greater than 0.500. ∆$r_{m}^{2}$, namely the absolute difference between $r_{m}^{2}$ and ${r^{\prime }}_{m}^{2}$, should be lower than 0.200 [47].

### 3.9. Virtual Screening

Topomer Search is an extremely fast ligand-based virtual screening tool that uses topomeric fields in addition to pharmacophoric properties to compare molecules [17]. In this work, R_1_ and R_2_ group fragments from the Topomer CoMFA model obtained were used as queries to search for R-groups that optimize activity. This search was performed in two stages. In the first stage, molecules in a database were broken into R-groups, which were compared with the two queries by Topomer similarity, respectively. In the second stage, R-groups, which passed the Topomer similarity cutoff (Topomer distance cutoff), were scored by their contribution to the predicted activity [46]. In this case, the ZINC Clean Drug-Like subset [50], which consists of approximately 13 million compounds was selected as the database for searching. Topomer distance cutoff was set as 185. The remaining parameters for Topomer Search were the default.

### 3.10. MD Simulations

The dynamics module of SYBYL-X 2.0 was utilized to perform MD simulations [26,34]. Two docked complexes—the complex of 1H1S with the newly designed **I13** and the complex of 1H1S with the newly designed **I60**, respectively, were used as the initial structures for MD simulations. The MMFF94 force field was used for the complexes and the atomic charges were set as the corresponding MMFF94 charges [51]. The canonical NVT (constant number of particles N, constant volume V, and constant temperature T) ensemble was used for the whole MD simulations. During the simulations, the temperature was kept at 300 K with a temperature coupling constant of 100 fs by using the Berendsen algorithm [52]. Initial atomic velocities were assigned by a Boltzmann distribution, which were consistent with the given temperature. The remaining parameters were at default. To guarantee the stability of each complex system, MD simulations were performed for 10 ns with a time step of 1 fs and the geometry and energy of each complex were recorded every 2 ps throughout the simulations.

## 4. Conclusions

3D-QSAR, virtual screening, molecular design, molecular docking, and MD simulations were carried out on a series of CDK2 inhibitors. The optimal CoMFA model (q^2^ = 0.743, $r_{\mathrm{pred}}^{2}$ = 0.991), CoMSIA model (q^2^ = 0.808, $r_{\mathrm{pred}}^{2}$ = 0.990), and Topomer CoMFA model (q^2^ = 0.779, $r_{\mathrm{pred}}^{2}$ = 0.962) showed good internal and external predictive capabilities. Analyses on contour maps of above 3D-QSAR models revealed that the following scenarios can enhance activity: adding positively charged substituents in R_1_, R_2_ and R_3_, adding hydrophilic substituents in R_3_, at the R_1_ position, introducing hydrophilic substituents in the region near the purine scaffold and introducing hydrophobic substituents in the region away from the purine scaffold, at the R_2_ position by introducing hydrophobic substituents in the region near the purine scaffold and introducing hydrophilic substituents in the region away from the purine scaffold, introducing the hydrogen bond donor and bulky substituents near the region where the amino group of the sulfonamide group at R_2_ position is located. In contrast, introducing steric bulk substituents in R_3_ will reduce the activity. Based on the results of R-group virtual screening, novel candidate compounds as potential CDK2 inhibitors were designed. 3D-QSAR model prediction results suggested that the 31 newly designed candidate compounds showed good predicted activity (predicted pIC_50_ > 8), which is comparable to that of template compound **9** (experimental pIC_50_ = 8.523). However, the candidate compounds’ selectivity toward other CDKs or similar kinases is not clear yet, which means that they may cause generalized cytotoxicity. Molecular docking results indicated that this series of inhibitors and the candidate compounds formed a conserved triplet of hydrogen bonds with the receptor in the binding site. All candidate compounds exhibited a Surflex–Dock total score of >8. Based on QSAR model prediction and molecular docking, two candidate compounds **I13** and **I60** (predicted pIC_50_ > 8, docking score > 10) were identified. The MD simulations of the complex **1H1S-I13** and **1H1S-I60** further confirmed their stability. In addition, molecular docking and MD simulations consistently indicated that the sulfonamide group of ligand and the residue Asp86 of receptor have a significant impact on activity. The Asp86, Glu81, Leu83, Lys89, Lys33, and Gln131 are important residues that can form hydrogen bonds with the ligand and affect the stability of the complex and activity of the ligand. This study could provide important guidance for the development of novel potential CDK2 inhibitors especially the candidate compounds **I13** and **I60**, which showed the greatest potential as target compounds.

## Figures and Tables

**Table 1:** The $r_{m}^{2}$ metrics values of the selected models.

Alignment	Model	${r_{m}^{2}}_{\left(\mathrm{overall}\right)}$	${{r^{\prime }}_{m}^{2}}_{\left(\mathrm{overall}\right)}$	${r_{m}^{2}}_{\left(\mathrm{test}\right)}$	${{r^{\prime }}_{m}^{2}}_{\left(\mathrm{test}\right)}$
1	CoMFA-SE	0.866	0.865	0.902	0.901
1	CoMSIA-HSE	0.857	0.855	0.897	0.891
1	CoMSIA-AHSE	0.841	0.840	0.906	0.905
2	CoMFA-SE	0.876	0.875	0.867	0.866
2	CoMSIA-DHSE	0.850	0.849	0.949	0.945
2	CoMSIA-AHSE	0.833	0.831	0.927	0.925
3	CoMSIA-ASE	0.816	0.817	0.829	0.856
3	CoMSIA-DHS	0.764	0.766	0.615	0.648
3	CoMSIA-DHSE	0.839	0.840	0.743	0.754
3	CoMSIA-AHSE	0.815	0.816	0.791	0.823
3	CoMSIA-DAHSE	0.831	0.832	0.806	0.831

**Table 2:** The validation statistical results for the optimal CoMFA, CoMSIA, and Topomer CoMFA models.

Parameter	CoMFA	CoMSIA	Topomer CoMFA
$r_{\mathrm{pred}}^{2}$	0.991	0.990	0.962
$R_{\mathrm{test}}^{2}$	0.991	0.994	0.971
$R_{0}^{2}$	0.999	0.996	0.992
${R^{\prime }}_{0}^{2}$	0.999	0.997	0.992
($R_{\mathrm{test}}^{2}$ − $R_{0}^{2}$)/$R_{\mathrm{test}}^{2}$	−0.008	−0.002	−0.022
($R_{\mathrm{test}}^{2}$ − ${R^{\prime }}_{0}^{2}$)/$R_{\mathrm{test}}^{2}$	−0.008	−0.003	−0.022
k	0.994	0.987	0.980
k’	1.006	1.013	1.019
MAE_(test)_	0.127	0.101	0.258
MAE_(train)_	0.151	0.154	0.295
σ_(test)_	0.054	0.105	0.113
σ_(train)_	0.121	0.155	0.229
$r_{m}^{2}$_(test)_	0.902	0.949	0.831
${r^{\prime }}_{m}^{2}$_(test)_	0.901	0.945	0.830
$r_{m}^{2}$_(avg)_	0.902	0.947	0.831
∆$r_{m}^{2}$_(test)_	0.001	0.004	0.001

**Table 3:** The actual and predicted pIC_50_ values of the dataset compounds.

Compound	pIC_50_	CoMFA		CoMSIA		Topomer CoMFA	
		Pred.	Res.	Pred.	Res.	Pred.	Res.
**1**	4.770	4.570	0.200	4.530	0.240	4.815	−0.045
2 *	6.013	6.239	−0.226	6.323	−0.310	6.311	−0.298
**3**	8.301	8.158	0.143	8.314	−0.013	8.476	−0.175
**4**	2.620	2.646	−0.026	2.429	0.191	2.562	0.058
**5**	4.215	4.191	0.024	4.181	0.034	4.057	0.158
**6**	5.824	5.662	0.162	5.910	−0.086	6.222	−0.398
7 *	8.097	8.260	−0.163	8.081	0.016	8.378	−0.281
**8**	8.000	8.161	−0.161	8.067	−0.067	8.212	−0.212
**9**	8.523	8.600	−0.077	8.472	0.051	8.389	0.134
10 *	7.721	7.575	0.146	7.782	−0.061	8.172	−0.451
**11**	7.770	7.582	0.188	7.511	0.259	7.740	0.030
12 *	4.678	4.807	−0.129	4.869	−0.191	4.889	−0.211
**13**	6.081	6.302	−0.221	6.614	−0.533	7.054	−0.973
14 *	5.194	5.107	0.087	5.303	−0.109	4.946	0.248
**15**	5.051	5.222	−0.171	5.137	−0.086	5.209	−0.158
**16**	6.658	7.002	−0.344	6.808	−0.150	6.972	−0.314
**17**	7.721	7.288	0.433	6.971	0.750	7.064	0.657
**18**	7.620	7.152	0.468	7.458	0.162	6.995	0.625
**19**	7.409	7.460	−0.051	7.690	−0.281	6.909	0.500
20 *	7.284	7.335	−0.051	7.271	0.013	7.006	0.278
**21**	7.357	7.481	−0.124	7.260	0.097	6.700	0.657
**22**	6.237	6.547	−0.310	6.313	−0.076	6.442	−0.205
**23**	5.854	5.948	−0.094	5.936	−0.082	5.651	0.203
**24**	5.174	5.142	0.032	5.264	−0.090	5.746	−0.572
**25**	4.350	4.449	−0.099	4.528	−0.178	4.619	−0.269
26 *	4.519	4.435	0.084	4.527	−0.008	4.561	−0.042
**27**	4.558	4.466	0.092	4.592	−0.034	4.554	0.004
**28**	4.046	4.064	−0.018	4.163	−0.117	4.314	−0.268
**29**	4.770	4.750	0.020	4.643	0.127	4.529	0.241
**30**	4.740	4.707	0.033	4.634	0.106	4.597	0.143
**31**	3.983	3.751	0.232	3.971	0.012	3.723	0.260
**32**	4.629	4.775	−0.146	4.849	−0.220	4.599	0.030
**33**	4.400	4.424	−0.024	4.336	0.064	4.606	−0.206
**34**	5.061	5.317	−0.256	5.197	−0.136	5.401	−0.340
**35**	6.292	6.200	0.092	6.234	0.058	5.857	0.435

* Test set compound. Pred.: predicted pIC_50_; Res.: residual.

**Table 4:**
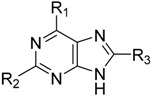
The structures and the pIC_50_ values of the dataset compounds.

Compound	R_1_	R_2_	R_3_	IC_50_ (µM) or % Inhibition	pIC_50_
**1**	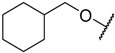	–NH_2_	–H	17.000	4.770
2 *	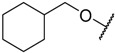	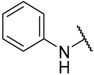	–H	0.970	6.013
**3**	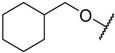	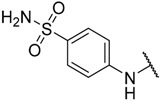	–H	0.005	8.301
**4**	–H	–NH_2_	–H	4% ^a^	2.620
**5**	–H	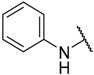	–H	61.000	4.215
**6**	–H	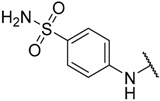	–H	1.500	5.824
7 *	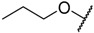	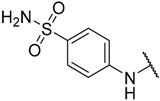	–H	0.008	8.097
**8**	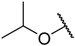	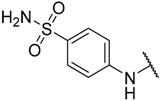	–H	0.010	8.000
**9**	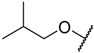	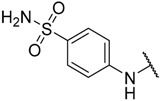	–H	0.003	8.523
10 *	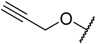	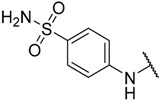	–H	0.019	7.721
**11**	–C≡CSi(*i*-Pr)_3_	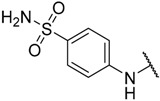	–H	0.017	7.770
12 *	–C≡CH	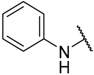	–H	21.000	4.678
**13**	–C≡CH	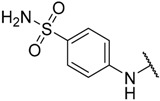	–H	0.830	6.081
14 *	–C≡CMe	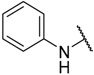	–H	6.400	5.194
**15**	–C≡CPh	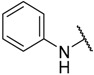	–H	8.900	5.051
**16**	–Et	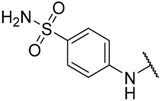	–H	0.220	6.658
**17**	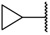	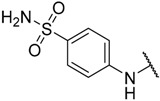	–H	0.019	7.721
**18**	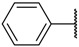	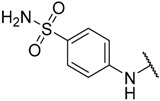	–H	0.024	7.620
**19**	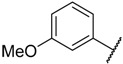	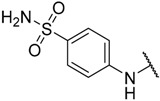	–H	0.039	7.409
20 *	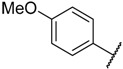	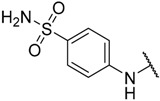	–H	0.052	7.284
**21**	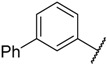	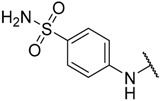	–H	0.044	7.357
**22**	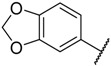	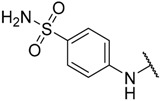	–H	0.580	6.237
**23**	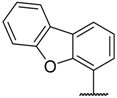	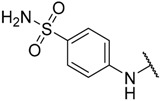	–H	1.400	5.854
**24**	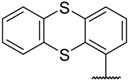	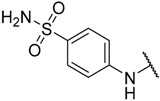	–H	6.700	5.174
**25**	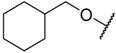	–NH_2_	–Me	44.700	4.350
26 *	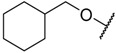	–NH_2_	–Et	30.300	4.519
**27**	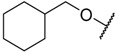	–NH_2_	–*i*-Pr	27.700	4.558
**28**	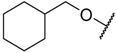	–NH_2_	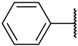	10% ^b^	4.046
**29**	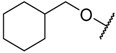	–NH_2_	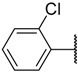	17.000	4.770
**30**	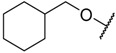	–NH_2_	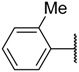	18.200	4.740
**31**	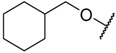	–NH_2_	–CF_3_	49% ^a^	3.983
**32**	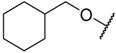	–NH_2_	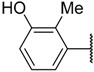	23.500	4.629
**33**	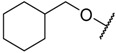	–NH_2_	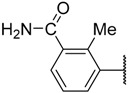	39.800	4.400
**34**	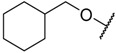	–NH_2_	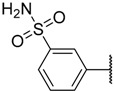	8.700	5.061
**35**	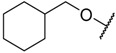	–NH_2_	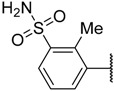	0.510	6.292

* Test set compound. ^a^ Activity measured at 100 μM. ^b^ Activity determined at 10 μM due to limiting solubility. Compounds **1**–**24** were collected from Reference [13]. Compounds **25**–**35** were collected from Reference [12].

## Supplementary Materials

The following are available online. Figure S1: Contour maps of the Topomer CoMFA model, Table S1: The PLS statistical results of CoMFA and CoMSIA models, Table S2: The structures, predicted pIC_50_ values of the newly designed candidate compounds, Table S3: Docking results of the dataset compounds, Table S4: Docking results of the newly designed candidate compounds.
