# Supplementary material for: Molecular Modeling and Design Studies of Purine Derivatives as Novel CDK2 Inhibitors
Source: Molecules. 2018 Nov 9;23(11):2924. doi: 10.3390/molecules23112924 (PMC6278423; doi:10.3390/molecules23112924)
Supplement: Supplementary file 1 [file molecules-23-02924-s001.pdf]

molecules

Supplementary Materials

Molecular Modeling and Design Studies of Purine Derivatives as Novel CDK2 Inhibitors

Gaomin Zhang and Yujie Ren *

School of Chemical and Environmental Engineering, Shanghai Institute of Technology, Shanghai 201418, China; gaomin.zhang@hotmail.com
* Correspondence: clab@sit.edu.cn

Table of Contents

Figure S1. Contour maps of the Topomer CoMFA model.

Table S1. The PLS statistical results of CoMFA and CoMSIA models.

Table S2. The structures, predicted pIC_{50} values of the newly designed candidate compounds.

Table S3. Docking results of the dataset compounds.

Table S4. Docking results of the newly designed candidate compounds.

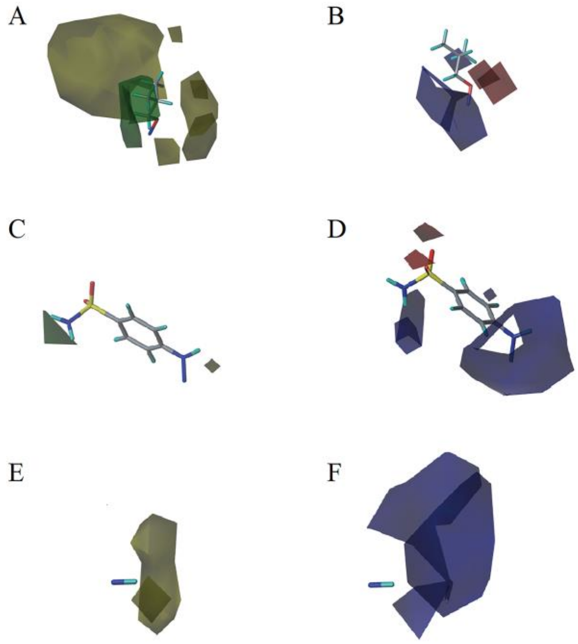

Figure S1. Contour maps of the Topomer CoMFA model. (**A**) R_1 steric contour; (**B**) R_1 electrostatic contour; (**C**) R_2 steric contour; (**D**) R_2 electrostatic contour; (**E**) R_3 steric contour; (**F**) R_3 electrostatic contour. Sterically favored (green) and disfavored (yellow). Electropositive favored (blue) and electronegative favored (red).

Table S1. The PLS statistical results of CoMFA and CoMSIA models.

Alignment	Model	q^2	ONC	SEE	r^2	*F*	Field Contribution				
							D	A	H	S	E
1	CoMFA-SE	0.743	5	0.219	0.984	273.426	-	-	-	0.577	0.423
1	CoMSIA-S	0.770	4	0.422	0.938	87.275	-	-	-	1.000	-
1	CoMSIA-E	0.750	5	0.394	0.948	81.017	-	-	-	-	1.000
1	CoMSIA-H	0.743	5	0.316	0.967	128.543	-	-	1.000	-	-
1	CoMSIA-A	0.652	2	0.829	0.741	35.745	-	1.000	-	-	-
1	CoMSIA-D	0.584	2	0.818	0.748	37.042	1.000	-	-	-	-
1	CoMSIA-SE	0.793	5	0.266	0.977	183.483	-	-	-	0.433	0.567
1	CoMSIA-DS	0.712	2	0.647	0.843	66.899	0.560	-	-	0.440	-
1	CoMSIA-AS	0.741	5	0.407	0.945	75.794	-	0.388	-	0.612	-
1	CoMSIA-HS	0.782	5	0.299	0.970	144.400	-	-	0.695	0.305	-
1	CoMSIA-DE	0.721	4	0.431	0.936	83.565	0.449	-	-	-	0.551
1	CoMSIA-AE	0.775	5	0.400	0.947	78.541	-	0.393	-	-	0.607
1	CoMSIA-HE	0.797	5	0.259	0.978	193.772	-	-	0.595	-	0.405
1	CoMSIA-DA	0.637	2	0.819	0.747	36.992	0.437	0.563	-	-	-
1	CoMSIA-DH	0.695	2	0.651	0.840	65.715	0.420	-	0.580	-	-
1	CoMSIA-AH	0.700	2	0.680	0.826	59.249	-	0.483	0.517	-	-
1	CoMSIA-HSE	0.805	5	0.233	0.982	240.400	-	-	0.458	0.208	0.334
1	CoMSIA-DSE	0.768	4	0.314	0.966	162.760	0.329	-	-	0.283	0.388
1	CoMSIA-ASE	0.796	5	0.290	0.972	153.934	-	0.277	-	0.321	0.403
1	CoMSIA-DHS	0.727	4	0.398	0.945	99.221	0.270	-	0.503	0.227	-
1	CoMSIA-AHS	0.751	5	0.325	0.965	121.608	-	0.216	0.539	0.246	-
1	CoMSIA-DAS	0.697	2	0.692	0.820	56.837	0.313	0.413	-	0.274	-
1	CoMSIA-DHE	0.783	4	0.321	0.964	155.174	0.250	-	0.437	-	0.313
1	CoMSIA-AHE	0.800	5	0.284	0.973	160.270	-	0.215	0.461	-	0.323
1	CoMSIA-DAE	0.741	4	0.433	0.935	82.960	0.304	0.242	-	-	0.454
1	CoMSIA-DAH	0.694	2	0.676	0.828	60.152	0.258	0.343	0.399	-	-
1	CoMSIA-DASE	0.775	5	0.283	0.973	161.186	0.232	0.187	-	0.244	0.337
1	CoMSIA-DHSE	0.796	5	0.229	0.983	247.958	0.204	-	0.357	0.165	0.274
1	CoMSIA-DAHE	0.781	5	0.283	0.974	161.863	0.179	0.152	0.381	-	0.288
1	CoMSIA-DAHS	0.719	5	0.316	0.967	128.484	0.185	0.151	0.456	0.208	-
1	CoMSIA-AHSE	0.810	5	0.259	0.978	192.821	-	0.189	0.371	0.171	0.270
1	CoMSIA-DAHSE	0.791	5	0.235	0.982	235.322	0.157	0.132	0.316	0.147	0.247
2	CoMFA-SE	0.746	5	0.192	0.988	357.035	-	-	-	0.618	0.382
2	CoMSIA-S	0.774	4	0.426	0.937	85.627	-	-	-	1.000	-
2	CoMSIA-E	0.779	5	0.392	0.949	82.186	-	-	-	-	1.000
2	CoMSIA-H	0.728	5	0.328	0.964	119.335	-	-	1.000	-	-
2	CoMSIA-A	0.649	2	0.833	0.738	35.273	-	1.000	-	-	-
2	CoMSIA-D	0.592	2	0.816	0.749	37.295	1.000	-	-	-	-
2	CoMSIA-SE	0.805	5	0.278	0.974	167.280	-	-	-	0.423	0.577
2	CoMSIA-DS	0.718	5	0.362	0.956	96.641	0.480	-	-	0.520	-
2	CoMSIA-AS	0.747	5	0.408	0.945	75.335	-	0.362	-	0.638	-
2	CoMSIA-HS	0.777	5	0.306	0.969	137.337	-	-	0.688	0.312	-
2	CoMSIA-DE	0.748	4	0.416	0.940	89.994	0.453	-	-	-	0.547
2	CoMSIA-AE	0.792	5	0.402	0.946	77.699	-	0.387	-	-	0.613
2	CoMSIA-HE	0.799	5	0.278	0.974	167.662	-	-	0.573	-	0.427
2	CoMSIA-DA	0.638	2	0.820	0.747	36.845	0.450	0.550	-	-	-
2	CoMSIA-DH	0.697	2	0.654	0.839	65.139	0.428	-	0.572	-	-
2	CoMSIA-AH	0.697	2	0.685	0.823	58.154	-	0.478	0.522	-	-
2	CoMSIA-HSE	0.807	5	0.253	0.979	203.055	-	-	0.438	0.210	0.353
2	CoMSIA-DSE	0.790	4	0.314	0.966	162.806	0.348	-	-	0.264	0.388
2	CoMSIA-ASE	0.812	5	0.293	0.971	149.812	-	0.276	-	0.313	0.411

Table S1. *Cont.*

Alignment	Model	q^2	ONC	SEE	r^2	*F*	Field Contribution				
							D	A	H	S	E
2	CoMSIA-DHS	0.730	4	0.406	0.943	94.789	0.283	-	0.489	0.228	-
2	CoMSIA-AHS	0.746	5	0.335	0.963	114.166	-	0.218	0.531	0.251	-
2	CoMSIA-DAS	0.706	5	0.411	0.944	74.184	0.356	0.226	-	0.418	-
2	CoMSIA-DHE	0.804	4	0.315	0.966	161.919	0.265	-	0.409	-	0.326
2	CoMSIA-AHE	0.807	4	0.338	0.960	139.492	-	0.241	0.433	-	0.326
2	CoMSIA-DAE	0.765	4	0.416	0.940	89.923	0.314	0.226	-	-	0.459
2	CoMSIA-DAH	0.694	2	0.678	0.827	59.683	0.267	0.333	0.400	-	-
2	CoMSIA-DASE	0.795	5	0.289	0.972	154.698	0.249	0.179	-	0.236	0.336
2	CoMSIA-DHSE	0.808	5	0.246	0.980	214.108	0.221	-	0.335	0.164	0.280
2	CoMSIA-DAHE	0.795	5	0.299	0.970	144.261	0.194	0.154	0.358	-	0.294
2	CoMSIA-DAHS	0.718	5	0.324	0.965	121.972	0.197	0.145	0.446	0.212	-
2	CoMSIA-AHSE	0.816	5	0.274	0.975	172.902	-	0.193	0.353	0.170	0.285
2	CoMSIA-DAHSE	0.804	4	0.312	0.966	165.253	0.172	0.145	0.295	0.143	0.245
3	CoMFA-SE	0.652	5	0.192	0.988	357.047	-	-	-	0.444	0.556
3	CoMSIA-S	0.359	5	0.551	0.899	39.327	-	-	-	1.000	-
3	CoMSIA-E	0.678	5	0.356	0.958	100.464	-	-	-	-	1.000
3	CoMSIA-H	0.530	5	0.362	0.957	97.152	-	-	1.000	-	-
3	CoMSIA-A	0.682	2	0.771	0.776	43.294	-	1.000	-	-	-
3	CoMSIA-D	0.601	2	0.739	0.794	48.275	1.000	-	-	-	-
3	CoMSIA-SE	0.770	5	0.207	0.986	306.604	-	-	-	0.286	0.714
3	CoMSIA-DS	0.801	5	0.278	0.974	166.928	0.610	-	-	0.390	-
3	CoMSIA-AS	0.747	5	0.370	0.955	92.804	-	0.604	-	0.396	-
3	CoMSIA-HS	0.601	5	0.368	0.955	93.399	-	-	0.660	0.340	-
3	CoMSIA-DE	0.670	5	0.349	0.960	104.296	0.403	-	-	-	0.597
3	CoMSIA-AE	0.721	5	0.309	0.968	134.927	-	0.377	-	-	0.623
3	CoMSIA-HE	0.724	5	0.232	0.982	242.689	-	-	0.423	-	0.577
3	CoMSIA-DA	0.656	2	0.738	0.795	48.442	0.474	0.526	-	-	-
3	CoMSIA-DH	0.706	5	0.233	0.982	240.208	0.487	-	0.513	-	-
3	CoMSIA-AH	0.711	5	0.288	0.972	155.480	-	0.431	0.569	-	-
3	CoMSIA-HSE	0.725	5	0.198	0.987	332.940	-	-	0.330	0.183	0.487
3	CoMSIA-DSE	0.784	5	0.206	0.986	307.447	0.339	-	-	0.225	0.436
3	CoMSIA-ASE	0.805	5	0.214	0.985	285.843	-	0.307	-	0.217	0.475
3	CoMSIA-DHS	0.764	5	0.193	0.988	351.809	0.429	-	0.365	0.206	-
3	CoMSIA-AHS	0.763	5	0.260	0.978	192.306	-	0.377	0.427	0.196	-
3	CoMSIA-DAS	0.793	5	0.315	0.967	129.599	0.388	0.299	-	0.314	-
3	CoMSIA-DHE	0.725	5	0.207	0.986	304.674	0.305	-	0.323	-	0.372
3	CoMSIA-AHE	0.768	5	0.241	0.981	223.552	-	0.285	0.333	-	0.383
3	CoMSIA-DAE	0.697	5	0.345	0.961	107.395	0.285	0.245	-	-	0.470
3	CoMSIA-DAH	0.720	5	0.271	0.976	176.841	0.311	0.256	0.433	-	-
3	CoMSIA-DASE	0.801	5	0.219	0.984	272.278	0.237	0.212	-	0.191	0.360
3	CoMSIA-DHSE	0.754	5	0.166	0.991	477.317	0.281	-	0.251	0.145	0.323
3	CoMSIA-DAHE	0.759	5	0.224	0.983	260.056	0.216	0.196	0.284	-	0.304
3	CoMSIA-DAHS	0.791	5	0.219	0.984	272.260	0.283	0.231	0.322	0.164	-
3	CoMSIA-AHSE	0.800	5	0.208	0.986	303.920	-	0.259	0.266	0.138	0.337
3	CoMSIA-DAHSE	0.787	5	0.189	0.988	369.031	0.205	0.183	0.225	0.123	0.264

q^2 is the square of the leave-one-out crossvalidation (LOO-CV) coefficient; ONC is the optimal number of components; SEE is the standard error of estimate; r^2 is the square of the non LOO-CV coefficient; *F* is the *F*-test value; D, A, H, S and E are the hydrogen bond donor, hydrogen bond acceptor, hydrophobic, steric, and electrostatic field contributions, respectively.

Table S2. The structures, predicted pIC_{50} values of the newly designed candidate compounds.

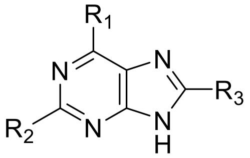

Compound	R_1	R_2	R_3	Predicted pIC_{50} CoMFA	Predicted pIC_{50} CoMSIA	Predicted pIC_{50} Topomer CoMFA
**I13**	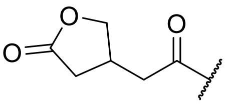	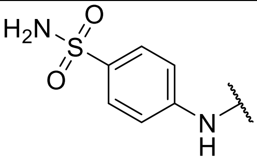	–H	9.417	8.274	9.310
**I19**	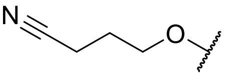	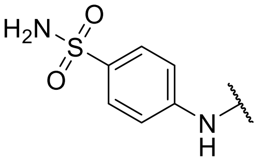	–H	8.855	8.441	8.866
**I21**	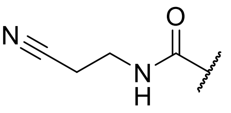	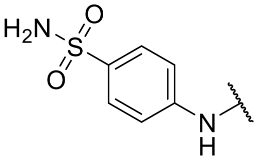	–H	8.526	8.333	9.192
**I33**	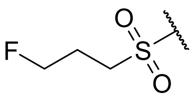	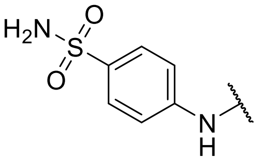	–H	8.488	8.180	9.050
**I34**	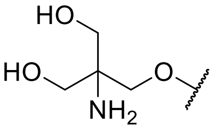	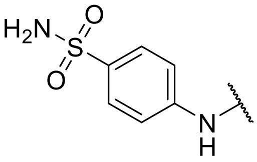	–H	8.656	8.209	8.810
**I36**	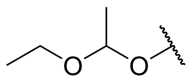	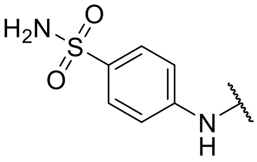	–H	8.101	8.487	8.739
**I38**	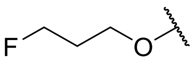	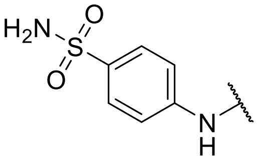	–H	8.554	8.413	8.655
**I39**	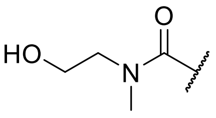	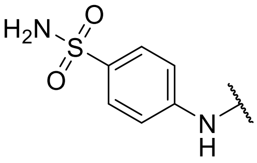	–H	8.490	8.201	9.030
**I40**	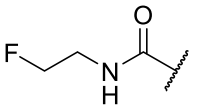	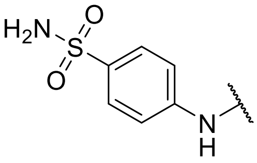	–H	8.335	8.150	8.998

Table S2. *Cont.*

Compound	R1	R2	R3	Predicted pIC50		
				CoMFA	CoMSIA	Topomer CoMFA
**I44**	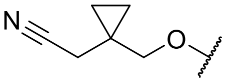	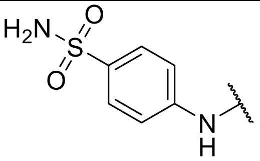	–H	8.578	8.027	8.751
**I46**	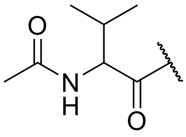	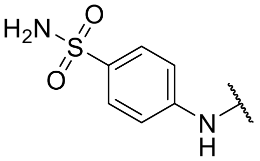	–H	8.163	8.107	9.041
**I52**	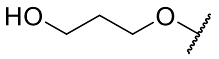	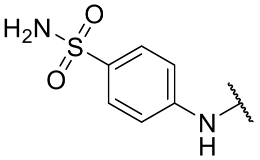	–H	8.182	8.294	8.579
**I59**	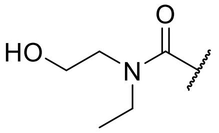	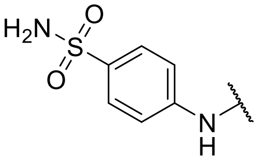	–H	8.433	8.124	8.950
**I60**	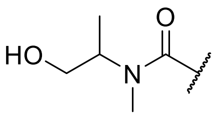	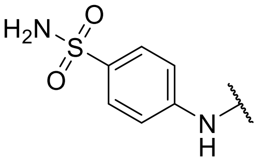	–H	8.304	8.292	8.935
**I62**	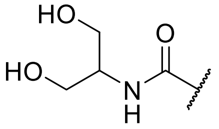	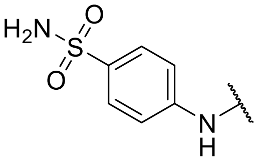	–H	8.779	8.304	8.908
**I63**	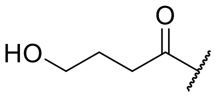	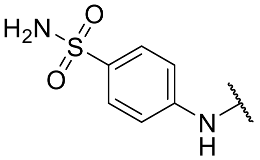	–H	8.396	8.389	8.896
**I66**	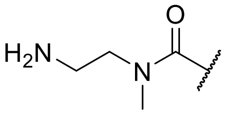	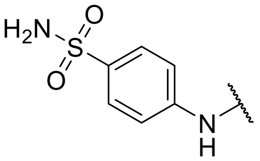	–H	8.416	8.228	8.912
**I67**	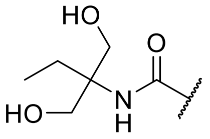	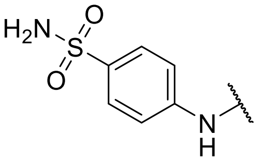	–H	8.785	8.253	8.898
**I155**	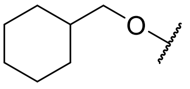	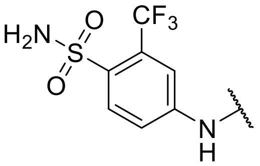	–H	8.180	8.231	8.673

Table S2. *Cont.*

Compound	R1	R2	R3	Predicted pIC_{50}		
				CoMFA	CoMSIA	Topomer CoMFA
**I173**	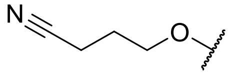	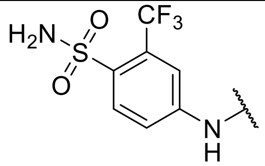	–H	8.736	8.234	9.064
**I178**	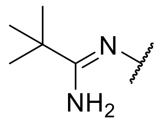	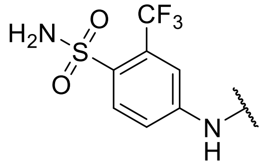	–H	8.450	8.068	8.424
**I190**	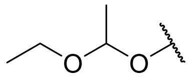	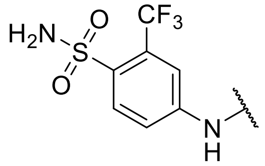	–H	8.592	8.282	8.937
**I192**	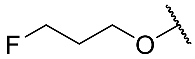	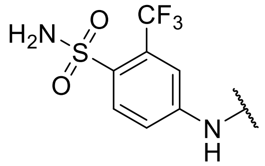	–H	8.768	8.211	8.858
**I193**	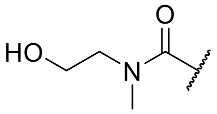	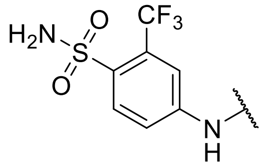	–H	8.348	8.243	9.228
**I206**	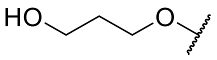	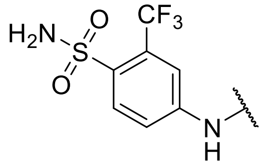	–H	8.460	8.061	8.777
**I213**	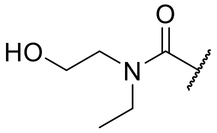	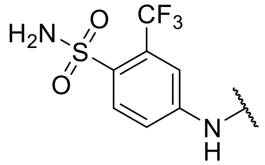	–H	8.122	8.170	9.148
**I214**	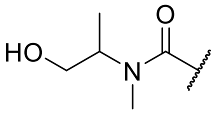	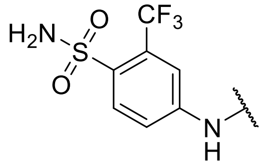	–H	8.660	8.250	9.133
**I215**	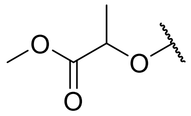	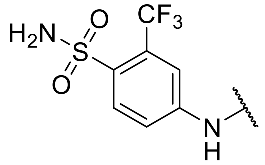	–H	8.556	8.050	8.795
**I217**	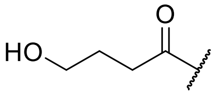	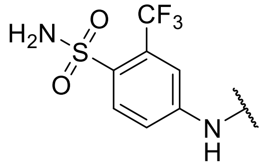	–H	8.531	8.098	9.094

Table S2. *Cont.*

Compound	R_1	R_2	R_3	Predicted pIC_{50}		
				CoMFA	CoMSIA	Topomer CoMFA
**I220**	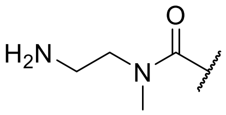	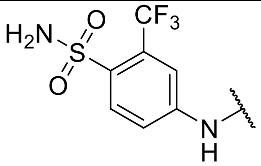	–H	8.257	8.268	9.110
**I225**	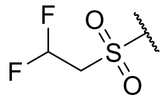	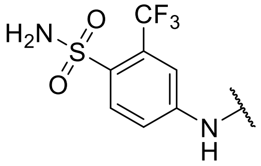	–H	8.685	8.024	9.058

Table S3. Docking results of the dataset compounds.

Compound	Total Score	Similarity	Cscore
**1**	8.439	0.670	2
**2 ***	8.893	0.727	3
**3**	11.128	0.894	5
**4**	5.644	0.576	4
**5**	4.946	0.425	5
**6**	7.592	0.816	5
**7 ***	9.516	0.851	4
**8**	8.886	0.836	4
**9**	10.319	0.865	4
**10 ***	8.574	0.724	3
**11**	10.707	0.672	2
**12 ***	6.509	0.655	5
**13**	8.222	0.722	5
**14 ***	7.080	0.663	5
**15**	6.890	0.684	5
**16**	8.204	0.718	4
**17**	8.466	0.790	5
**18**	9.070	0.859	5
**19**	10.224	0.675	4
**20 ***	9.743	0.851	5
**21**	9.404	0.838	3
**22**	10.299	0.841	5
**23**	9.310	0.860	4
**24**	6.768	0.756	5
**25**	8.783	0.567	3
**26 ***	9.139	0.606	3
**27**	9.916	0.577	4
**28**	9.258	0.600	1
**29**	9.376	0.616	2
**30**	10.027	0.562	2
**31**	8.893	0.575	4
**32**	10.671	0.545	5
**33**	10.591	0.697	1
**34**	11.475	0.751	1
**35**	11.956	0.752	1

* Test set compound. Total score is the total Surflex–Dock score expressed as –log(K_d); Similarity is the Surflex–Sim similarity between the top scoring pose of compound and the cognate ligand; Cscore is the consensus score.

Table S4. Docking results of the newly designed candidate compounds.

Compound	Total Score	Similarity	Cscore
**I13**	11.734	0.813	5
**I19**	9.070	0.699	5
**I21**	9.487	0.843	5
**I33**	10.074	0.828	4
**I34**	10.379	0.678	3
**I36**	10.068	0.748	4
**I38**	10.019	0.760	3
**I39**	8.219	0.796	5
**I40**	9.905	0.843	4
**I44**	10.188	0.851	4
**I46**	11.183	0.701	2
**I52**	8.283	0.720	3
**I59**	11.052	0.790	5
**I60**	10.057	0.821	5
**I62**	9.748	0.775	4
**I63**	10.422	0.694	2
**I66**	8.756	0.709	5
**I67**	10.790	0.796	4
**I155**	11.569	0.844	4
**I173**	9.991	0.680	2
**I178**	9.882	0.783	5
**I190**	11.175	0.826	4
**I192**	8.443	0.691	5
**I193**	10.139	0.760	5
**I206**	9.788	0.666	3
**I213**	10.601	0.794	4
**I214**	9.154	0.693	3
**I215**	10.046	0.765	4
**I217**	9.964	0.771	5
**I220**	10.478	0.645	3
**I225**	9.782	0.788	4

Total score is the total Surflex–Dock score expressed as –log(K_d); Similarity is the Surflex–Sim similarity between the top scoring pose of compound and the cognate ligand; Cscore is the consensus score.
